# Radar Constant-Modulus Waveform Design with Prior Information of the Extended Target and Clutter

**DOI:** 10.3390/s16060889

**Published:** 2016-06-17

**Authors:** Wenzhen Yue, Yan Zhang, Yimin Liu, Jingwen Xie

**Affiliations:** 1 Beijing Institute of Tracking and Telecommunications Technology (BITTT), Beijing 100094, China; zhyan1227@sina.com (Y.Z.); jackyue1994@163.com (J.X.); 2 Department of Electronic Engineering, Tsinghua University, Beijing 100084, China; yiminliu@tsinghua.edu.cn

**Keywords:** radar waveform design, constant-modulus waveform, extended target detection, clutter, waveform optimization

## Abstract

Radar waveform design is of great importance for radar system performances and has drawn considerable attention recently. Constant modulus is an important waveform design consideration, both from the point of view of hardware realization and to allow for full utilization of the transmitter’s power. In this paper, we consider the problem of constant-modulus waveform design for extended target detection with prior information about the extended target and clutter. At first, we propose an arbitrary-phase unimodular waveform design method via joint transmitter-receiver optimization. We exploit a semi-definite relaxation technique to transform an intractable non-convex problem into a convex problem, which can then be efficiently solved. Furthermore, quadrature phase shift keying waveform is designed, which is easier to implement than arbitrary-phase waveforms. Numerical results demonstrate the effectiveness of the proposed methods.

## 1. Introduction

It has been widely acknowledged that waveform design is critically important for radar performance in areas such as target detection, clutter suppression, and target identification, especially when in the presence of channel noise and clutter [1,2]. However, optimal radar waveform is highly task-dependent and is also affected by the model of the target and surrounding environments. In the 1960s, some early research on radar waveform optimization was conducted [1,2,3,4,5]. All the early works assumed a point target model. However, as radar bandwidth and range resolution improve, the illuminated target usually exceeds one resolution cell. Thus, the point target assumption does not hold and should be replaced by an extended target model [6]. Therefore, the paper considers waveform design for extended targets. 

Generally, radar waveform design methods can be broken into four categories: (1) information theory based design [7,8,9,10,11,12,13,14]; (2) ambiguity-function based design [15,16,17]; (3) detection-probability based design [18,19,20]; and (4) signal-to-clutter-plus-noise ratio (SCNR) based design [21,22,23,24,25,26,27]. Information theoretic methods were inspired by the very essence of radar—acquiring target related information [7]. Mutual information (MI) criteria between the target return and the target impulse response (TIR) was later used for radar parameter estimation and target identification [8,9,10,11,12,13,14]. The relationship between minimum mean squared error and MI metrics was discussed in [9,11], and the case of multiple extended targets was considered in [12]. Ambiguity-function based methods [15,16,17] design the transmitted waveform by reshaping its ambiguity function—suppressing the Doppler-range (angle-Doppler-range, in the MIMO radar case) region where targets might emerge. These methods require prior information about the range and Doppler distribution of clutter and/or jamming—which is more than the statistical characteristics that would be needed in the detection-probability and SCNR based methods. However, the methods based on ambiguity functions deal mainly with point targets, which is somewhat off the topic of this paper.

The basic idea of detection-probability based methods is to optimize the transmitted waveform to maximize detection probability for a given false alarm rate. It has been shown that a simple single-tone signal is optimal if the target’s Doppler is unknown and is uniformly distributed over the Doppler bandwidth [18]. Steven Kay [19] obtained an analytical solution of the optimal waveform for Gaussian-distributed point target using the Neyman-Pearson criterion. The results in [19] were heuristically extended to the multistatic radar case [20]. However, detection probability based methods encounters difficulties with extended targets and waveform-dependent clutter, because in these cases the explicit probability density function of the radar return is hard to obtain, and further analysis becomes very difficult. Fortunately, we can resort to SCNR based methods, whose optimization criterion is to maximize the output SCNR. Bell derived an eigen-waveform solution for a clutter-free environment with energy constraints, for the first time [8]. However, in practice, radar returns are usually contaminated by clutter coupled with the transmitted waveform. This coupling poses a big challenge for optimal waveform design. To overcome the problem, Pillai [22,23] proposed an iterative algorithm—which is also called the *eigen-iterative* algorithm in some literature—to determine the optimal transmit pulse and receiver impulse response. This iterative technique was later extended to the discrete time domain [24] and the multiple-input multi-output (MIMO) radar case [26]. However, the technique in [22,23,24] is flawed, in that it cannot guarantee a non-decreasing SCNR at each iteration step. Chen *et al.* [26] solved this problem under a total energy constraint through alternate optimization, improving the SCNR at each iteration, and thus guaranteeing its convergence. 

However, much existing work [8,10,11,12,13,14,16,17,18,19,20,22,23,24,25,26,27] is based on the assumption that radar transmitted signals can be modulus-arbitrary—something which is still difficult to implement in present radar systems—limited by the linear range of the radio frequency (RF) power amplifier and the capabilities of present RF antennas [5,21,28,29]. Accordingly, the design of radar waveform with limited dynamic range was considered in [5,28], and constant-modulus waveform design was discussed in [21,29]. Note that constant-modulus waveform is important for power efficient radars, such as airborne and spaceborne radars. In [21], an adaptive phase-coded waveform design was proposed, but it did not take the clutter into account, which hampered its practicability. Reference [29] optimizes a phase-modulated waveform by approaching the optimal energy spectral density in the mean-square sense, but the method requires prior knowledge of the power spectral density of channel noise and clutter. In this paper, we further extend the design of constant-modulus waveforms in discrete time domain in the presence of clutter. 

As is well known, the TIR is highly range and orientation sensitive [6]. Small variations in target range or orientation relative to the radar might lead to significant TIR changes. Therefore, we think that a deterministic and precisely known TIR is not realistic. Instead, we consider the TIR in a statistical way. This assumption has also been made in [8,9,10,11,12,13,14,18,19,20,26,29,30]. Note that, as will be demonstrated later, our methods are suitable for the deterministic target model as well. In addition, we concentrate on single-input single-output (SISO) radar in this paper: the extension of our methods to the MIMO radar case is straightforward. 

The main contributions of this paper are summarized as follows. We propose two iterative constant-modulus waveform design methods, both using alternate optimization of the transmitted waveform and the receiving filter. One of these methods yields arbitrary-phase waveforms, while the other yields quadrature phase shift keying (QPSK) waveforms. We also discuss the relationship between a non-convex optimization problem and the corresponding convex problem that results after semi-definite relaxation (SDR), which could be instructive to similar optimization problems in signal processing fields. 

The rest of the paper is organized as follows. Section 2 presents the radar signal model and formulates the signal design problem. In Section 3, two iterative constant-modulus waveform design methods are proposed. Detailed performance analysis of our methods is provided in Section 4. In Section 5, we show the results of numerical simulations and demonstrate the effectiveness of our proposed algorithms. Finally, conclusions are presented in Section 6.

*Notations*: Vectors and matrices are denoted by boldface lowercase and uppercase letters, respectively. Superscript *^T^*, ^*^, and *^H^* denote transpose, conjugation and Hermitian transpose of a vector/matrix, respectively. $I_{n}$ is the n×n unity matrix, whereas $0_{m\times n}$ and $1_{m\times n}$ ($0_{n}$ and $1_{n}$) indicate m×n (n×1) matrices whose elements are all 0 and 1, respectively. The subscript may be omitted if it does not cause confusion in the matrix/vector size. $A_{mn}$ represents the element of matrix **A** located at the *m*th row and *n*th column. $z_{n}$ or z(n) represents the *n*th component of vector **z**. z(i:j) denotes the segment of z from the *i*th element to *j*th element. E(·) denotes the expected value of a random variable. We write Re(·), Im(·), and |⋅| for the real part, imaginary part and modulus of a complex scalar/matrix, respectively. $\lambda_{i}\left(A\right)$ denotes the *i*th largest eigenvalue of **A** and $v_{i}\left(A\right)$ denotes the corresponding eigenvector. A≽0 (A≻0) means that matrix **A** is Hermitian positive semi-definite (positive definite). A≽B means that A−B≽0. j is the imaginary unit. CN and N designate the complex and real normal distributions, respectively.

## 2. Signal Model and Problem Formulation

**Figure 1:**
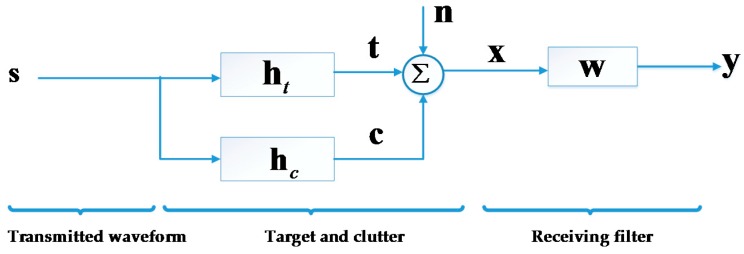
Illustration of the radar’s discrete baseband signal model.

In this paper, we use the discrete baseband signal model illustrated in Figure 1. In this figure, $s\in {\mathbb{C}}^{N_{s}\times 1}$denotes the transmitted waveform; $h_{t}\in {\mathbb{C}}^{N_{t}\times 1}$ and $h_{c}\in {\mathbb{C}}^{N_{c}\times 1}$ are the impulse response of target and clutter, respectively; $n\in {\mathbb{C}}^{N_{n}\times 1}$ denotes the sum of noise and interference/jamming with covariance matrix $R_{n}$; x denotes the returns from target and ambient clutter, while **w** denotes the receiving filter vector. In a practical radar system, s is converted to an analog waveform, modulated to RF, and transmitted. Inversely, the returns are received, demodulated and down-sampled to a discrete vector signal. In this paper, we focus on the discrete baseband signal in the time domain and do not discuss continuous signals or the frequency domain further.

As shown in Figure 1, we model the clutter return **c** as the output of a random linear time invariant (LTI) filter whose impulse response $h_{c}$ can be regarded as a wide sense stationary (WSS) random vector with covariance matrix $R_{c}$. As mentioned in Section 1, we consider $h_{t}$ in a statistical way [8,9,10,11,12,13,14,18,19,20,26,29,30] and its covariance matrix is denoted as $R_{t}$. We have assumed that the target is static (or equivalently, that the Doppler shift has been precisely measured and compensated), which is also implied in Figure 1. The assumption of the zero-Doppler target has also been made in [8,9,10,11,12,13,14,19,22,23,24,25,26,27,29]. According to [19], if we obtain the optimal waveform with this model, improved performance will result with the optimized waveform in the moving target case as well. We will explore the moving target case in the future.

According to our model, the returns can be formulated as:

(1) $$x=h_{t}*s+c+n$$

where * denotes the convolution operator. The matrix form of Equation (1) is:

(2) $$x=H_{t}s+H_{c}s+n$$

where $H_{t}$ and $H_{c}$ are the target and clutter convolution matrices, and are shown in Equations (3) and (4), respectively. Note that the differences between Equations (3) and (4) come from the ubiquity of the clutter, because anything in the illuminated area of the radar that does not interest us can be viewed as clutter [26]. From Equation (3), we obtain $H_{t}=\left[\xi_{0}\;\cdots \;\xi_{N_{s}-1}\right]$, where $\xi_{i}={\left[0_{i}^{T},\;h_{t}^{T},0_{N_{t}-i-1}^{T}\right]}^{T}$. We use function $H_{t}=f\left(h_{t},N_{s}\right)$ to represent the relationship between $H_{t}$ and $h_{t}$.

(3) $$H_{t}=\left[\begin{array}{cccc} h_{t}\left(1\right) & 0 & \cdots & 0\\ \begin{array}{c} \vdots \\ h_{t}\left(N_{t}\right)\\ 0 \end{array} & \begin{array}{c} h_{t}\left(1\right)\\ \vdots \\ h_{t}\left(N_{t}\right) \end{array} & \begin{array}{c} \ddots \\ \ddots \\ \ddots \end{array} & \begin{array}{c} \vdots \\ 0\\ h_{t}\left(1\right) \end{array}\\ \vdots & \ddots & \ddots & \vdots \\ 0 & \cdots & 0 & h_{t}\left(N_{t}\right) \end{array}\right]$$

(4) $$H_{c}=\left[\begin{array}{cccc} h_{c}\left(0\right) & h_{c}\left(-1\right) & \cdots & h_{c}\left(-N_{s}+1\right)\\ \begin{array}{c} h_{c}\left(1\right)\\ \vdots \\ \vdots \end{array} & \begin{array}{c} h_{c}\left(0\right)\\ h_{c}\left(1\right)\\ \ddots \end{array} & \begin{array}{c} \ddots \\ \ddots \\ \ddots \end{array} & \begin{array}{c} \vdots \\ \vdots \\ h_{c}\left(0\right) \end{array}\\ \begin{array}{c} \vdots \\ \vdots \end{array} & \begin{array}{c} \ddots \\ \ddots \end{array} & \begin{array}{c} \ddots \\ \ddots \end{array} & \begin{array}{c} h_{c}\left(1\right)\\ \vdots \end{array}\\ h_{c}\left(N_{t}+N_{s}-2\right) & \cdots & \cdots & h_{c}\left(N_{t}-1\right) \end{array}\right]$$

From Equation (4), we define vector $h_{c}$ as:

(5) $$h_{c}={\left[\begin{array}{ccc} h_{c}\left(-N_{s}+1\right) & \cdots & \begin{array}{ccc} h_{c}\left(0\right) & \cdots & h_{c}\left(N_{t}+N_{s}-2\right) \end{array} \end{array}\right]}^{T}$$

It is apparent that $N_{s}$, $N_{t}$, $N_{n}$ and $N_{c}$, which are the length of s, $h_{t}$, n and $h_{c}$, respectively, satisfy the following identities: 

(6) $$N_{c}=N_{t}+2N_{s}-2,\;\;\;N_{n}=N_{t}+N_{s}-1$$

Using Equation (2), the receiver output can be expressed as:

(7) $$y=w^{H}x=\underset{signal}{\underset{︸}{w^{H}H_{t}s}}+\underset{clutter\;and\;noise}{\underset{︸}{w^{H}\left(H_{c}s+n\right)}}$$

where the target return is $w^{H}H_{t}s$, and the sum of the clutter return and channel noise is $w^{H}\left(H_{t}s+n\right)$. Considering the statistical property of the clutter and target, it is necessary to take the expectation value of the clutter return and target return. Thus, the SCNR of the output signal can be written as:

(8) $$SCNR\;\;=\;\frac{E\left[{\left|w^{H}H_{t}s\right|}^{2}\right]}{E\left[{\left|w^{H}\left(H_{c}s+n\right)\right|}^{2}\right]}≜\frac{w^{H}R_{ts}w}{w^{H}\left(R_{cs}+R_{n}\right)w}≜\frac{s^{H}R_{tw}s}{s^{H}\left(R_{cw}+\frac{w^{H}R_{n}w}{N_{s}}\cdot I\right)s}$$

where, for notational simplicity, we let

(9) $$\left\{ \begin{array}{l} R_{ts}≜E\left[H_{t}ss^{H}H_{t}^{H}\right],\;\;\;\;\;\;\;\;R_{cs}≜E\left[H_{c}ss^{H}H_{c}^{H}\right]\\ R_{tw}≜E\left[H_{t}^{H}ww^{H}H_{t}\right],\;\;\;\;\;R_{cw}≜E\left[H_{c}{}^{H}ww^{H}H_{c}\right] \end{array}\right.$$

Define $S_{1}≜f\left(s,N_{t}\right)$, $S_{2}≜f\left(s,N_{c}\right)$, and ${\tilde{H}}_{c}≜f\left(h_{c},N_{s}\right)$. With the fact that $H_{t}s=S_{1}h_{t}$, we have

(10) $$R_{ts}=E\left[H_{t}ss^{H}H_{t}^{H}\right]=E\left[S_{1}h_{t}h_{t}{}^{H}S_{1}^{H}\right]=S_{1}R_{t}S_{1}^{H}$$

Letting $Q≜\left[0_{N_{n}\times \left(N_{s}-1\right)}\;I_{N_{n}\times N_{n}}\;0_{N_{n}\times \left(N_{s}-1\right)}\right]$, $H_{c}$ can then be written as $H_{c}=Q{\tilde{H}}_{c}$. It follows that

(11) $$R_{cs}=E\left[H_{c}ss^{H}H_{c}^{H}\right]=QE\left[{\tilde{H}}_{c}ss^{H}{\tilde{H}}_{c}^{H}\right]Q^{H}=QE\left[S_{2}h_{c}h_{c}{}^{H}S_{2}^{H}\right]Q^{H}=QS_{2}R_{c}S_{2}^{H}Q^{H}$$

Moreover, the definition of

(12) $$W≜{\left[\begin{array}{cccc} \zeta_{1} & \zeta_{2} & \cdots & \zeta_{N_{s}} \end{array}\right]}^{T},\;\;\zeta_{i}≜w\left(i:N_{t}+i-1\right)$$

produces $H_{t}^{H}w=Wh_{t}^{*}$, and

(13) $$R_{tw}=E\left[H_{t}^{H}ww^{H}H_{t}\right]=E\left[Wh_{t}{}^{*}h_{t}{}^{T}W^{H}\right]=WR_{t}{}^{*}W^{H}$$

Use $W=g\left(w,N_{t}\right)$ to represent the relationship between W and w in Equation (12). Define $\tilde{w}≜{\left[0_{N_{s}-1}^{T}\;w^{T}\;0_{N_{s}-1}^{T}\right]}^{T}$, and $\tilde{W}≜g\left(\tilde{w},N_{c}\right)$, then we have $H_{c}^{H}w={\tilde{H}}_{c}^{H}\tilde{w}=\tilde{W}h_{t}^{*}$, and

(14) $$R_{cw}=E\left[H_{c}{}^{H}ww^{H}H_{c}\right]=E\left[{\tilde{H}}_{c}^{H}\tilde{w}{\tilde{w}}^{H}{\tilde{H}}_{c}\right]=E\left[\tilde{W}h_{c}^{*}h_{c}{}^{T}{\tilde{W}}^{H}\right]=\tilde{W}R_{c}^{*}{\tilde{W}}^{H}$$

It should be noted that, even though Equations (8) and (10)–(14) are obtained based on the statistical target assumption, they also hold for the deterministic extended target. The only difference is that the rank of $R_{t}$ in the deterministic case will be 1. Equations (10)–(14) provide an easy analytical way to compute matrices $R_{ts}$, $R_{cs}$, $R_{tw}$, and $R_{cw}$, whereas the randomization method presented in [26] to compute these matrices suffers from inaccuracy and heavy computation. On the basis of Equations (8) and (10)–(14), our next goal is to optimize **s** under the constant-modulus constraint, to maximize SCNR.

## 3. Constant-Modulus Waveform Design Methods

In this section, two iterative constant-modulus signal design algorithms are proposed, whose key idea is to alternately optimize the transmitted waveform **s** and the receiving filter **w** to improve SCNR at each iteration step. We adopt the following strategy: first, we optimize **w** for fixed transmitted waveform **s**; then, we optimize **s** under the constant modulus constraint, for the fixed receiving filter **w** previously found. Even though the alternate optimization strategy may become trapped by local optima, it could at least achieve the optimum separately along the **w** dimension and **s** dimension at the “trapping” point [26,31].

### 3.1. Algorithm 1—Arbitrary-Phase Waveform Design

First, when **s** is fixed, the optimization problem becomes:

(15) $$\underset{w}{\max}\frac{w^{H}R_{ts}w}{w^{H}\left(R_{cs}+R_{n}\right)w}$$

This is the well-known Rayleigh quotient problem. The optimal solution for Equation (15) is:

(16) $$w_{opt}=v_{1}\left({\left(R_{cs}+R_{n}\right)}^{-1}R_{ts}\right)$$

and the maximum is $\lambda_{1}\left({\left(R_{cs}+R_{n}\right)}^{-1}R_{ts}\right)$. Substitution of Equation (16) back into Equation (15) yields an expression of SCNR as a function of **s**. However, determining the **s** that maximizes the SCNR using that expression is not simple. This is also why we take an alternate optimization strategy here.

When **w** is fixed, without the constant modulus constraint, the optimization problem turns out to be:

(17) $$\underset{s}{\max}\frac{s^{H}R_{tw}s}{s^{H}\left(R_{cw}+\frac{w^{H}R_{n}w}{N_{s}}\cdot I\right)s}$$

Similarly, the optimal solution is:

(18) $$s_{opt}=v_{1}\left({\left(R_{cw}+\frac{w^{H}R_{n}w}{N_{s}}\cdot I\right)}^{-1}R_{tw}\right)$$

with the maximum SCNR $\lambda_{1}\left({\left(R_{cw}+\frac{w^{H}R_{n}w}{N_{s}}\cdot I\right)}^{-1}R_{tw}\right)$. 

However, things become complicated if s is required to be constant-modulus. Recognizing that, in this case, the amplitude of s is simply a scale factor with no effect on SCNR, we henceforth assume s to be unimodular. To simplify the notation, we let $R_{tw}≜\Omega_{1}$, and $R_{cw}+\frac{w^{H}R_{n}w}{N_{s}}\cdot I≜\Omega_{2}$. The optimization problem Equation (17) can then be expressed as:

(19) $$\underset{s}{\max}\frac{s^{H}\Omega_{1}s}{s^{H}\Omega_{2}s}\;\;\;s.t.\;\;\left|s\left(k\right)\right|=1,\;\;k=1,\;\cdots ,\;N_{s}$$

Unfortunately, the optimization problem Equation (19) is nonconvex, and needs to be converted to a problem that can be efficiently solved. Letting $X≜ss^{H}$, we can rewrite Equation (19) as:

(20) $$\begin{array}{l} \underset{X}{\max}\frac{tr\left(\Omega_{1}X\right)}{tr\left(\Omega_{2}X\right)}\\ s.t.\;\;diag\left(X\right)=1_{N_{s}},\;\;rank\left(X\right)=1,\;X≽0 \end{array}$$

where tr(·) denote the trace of a matrix. As long as it does not lead to confusion, we henceforth omit the subscript $N_{s}$ of $1_{N_{s}}$ in Equation (20). However, Equation (20) still is a nonconvex problem owing to the non-convexity of both the objective function and the constraint condition rank(X)=1. Fortunately, the semi-definite relaxation (SDR) technique can remove the non-convexity of constraint rank(X)=1 by relaxing it into X≽0. The SDR technique and the following randomization approach are commonly used for optimization problems in the signal processing research area (e.g., [32], for recent literature). Applying SDR, Equation (20) then becomes:

(21) $$\begin{array}{l} \underset{X}{\max}\frac{tr\left(\Omega_{1}X\right)}{tr\left(\Omega_{2}X\right)}\\ s.t.\;\;diag\left(X\right)=1,\;X≽0 \end{array}$$

Lemma 1 then converts Equation (21) into an equivalent convex problem.

**Lemma 1.**  *If $X_{opt}$ and $t_{opt}$ solve the convex optimization problem*

(22) $$\begin{array}{l} \underset{X,t}{\min}tr\left(\Omega_{2}X\right)\\ s.t.\;\;tr\left(\Omega_{1}X\right)=1,\;diag\left(X\right)=t\cdot 1,\;X≽0 \end{array}$$

*then $X^{†}≜X_{opt}/t_{opt}$ solves Equation (21).*

**Proof.**  According to the definition of $X_{opt}$ and $t_{opt}$, we have

(23) $$X^{†}=X_{opt}/t_{opt}≽0,\;\;diag\left(X^{†}\right)=diag\left(X_{opt}\right)/t_{opt}=1$$

Hence, $X^{†}$ is within the feasible region of Equation (21).

For any matrix $X\in {\mathbb{C}}^{N_{s}\times N_{s}}$ which satisfies diag(X)=1, X≽0, we define $tr\left(\Omega_{1}X\right)≜a$, $X^{\prime }≜X/a$. We then have $tr\left(\Omega_{1}X^{\prime }\right)≜1$, and

(24) $$tr\left(\Omega_{2}X'\right)\geq tr\left(\Omega_{2}X_{opt}\right)$$

which derives from the fact that $X_{opt}$ is the optimal solution of Equation (22). It follows that

(25) $$\begin{array}{l} \frac{tr\left(\Omega_{1}X^{†}\right)}{tr\left(\Omega_{2}X^{†}\right)}=\frac{tr\left(\Omega_{1}X_{opt}\right)/t_{opt}}{tr\left(\Omega_{2}X_{opt}\right)/t_{opt}}\\ \;\;\;\;\;\;\;\;\;\;\;\;\;\;=\frac{tr\left(\Omega_{1}X_{opt}\right)}{tr\left(\Omega_{2}X_{opt}\right)}=\frac{1}{tr\left(\Omega_{2}X_{opt}\right)}\\ \;\;\;\;\;\;\;\;\;\;\;\;\;\;\geq \frac{1}{tr\left(\Omega_{2}X'\right)}=\frac{tr\left(\Omega_{1}X\right)}{tr\left(\Omega_{2}X\right)} \end{array}$$

which completes the proof.

Solving the convex problem Equation (22) with the existing toolboxes (e.g., CVX toolbox [33]) is much easier than solving Equation (21). However, it should be noted that the optimal value of the objective function in Equation (22) is lower than that of Equation (19). The approximation bound for the two optima will be discussed in the next section. To obtain the optimal signal $s_{opt}$ with the optimal solution $X^{†}$ of Equation (21), we propose a customized randomization approach, shown in Table 1.

One can notice that we use $s_{\left(can,k\right)}=exp\left(-j\cdot ang\left(s_{\left(k\right)}\right)\right)$, other than $s_{\left(can,k\right)}=exp\left(j\cdot ang\left(s_{\left(k\right)}\right)\right)$, to normalize $s_{k}$ in step 4 of Table 1. The reason is that, even though they perform similarly according to our numerical experiments, the former facilitates further analysis of the SDR technique in the next section. Please note that both forms perform well in our simulations. The procedure of the iterative arbitrary-phase waveform design method (*i.e.*, Algorithm 1) is presented in Table 2.

### 3.2. Algorithm 2—QPSK Waveform Design

In this subsection, we discuss the waveform design method using a QPSK waveform instead of an arbitrary-phase waveform. The main difference between these two methods lies in the way candidate vectors are generated. In [34], we showed that QPSK signal could approximate a given covariance matrix quite closely. The method in [34] is based on the change of correlation properties arising from memoryless nonlinear transformation of Gaussian process [35], and could be applied here. Consider two Gaussian variables $x_{m}$, $x_{n}$ with zero mean, unit variance, and covariance *ρ*. Then, the covariance γ of $\text{sgn}\left(x_{m}\right)$ and $\text{sgn}\left(x_{n}\right)$, where sgn(x) takes the value +1, −1 when x is positive and negative, respectively, satisfies [35]

(26) $$\rho =\sin\left(\frac{\pi }{2}\gamma \right)$$

This equation is the foundation of our QPSK waveform design. The candidate QPSK vector design procedure using matrix $X^{†}$, the optimal solution of Equation (21), is presented in Table 3. Further details can be found in [34]. 

Note that, in Step 3, we use a forced positive Cholesky decomposition because $\tilde{X}$ cannot be guaranteed to be semi-positive definite. We refer the interested readers to [34], where we presented one equivalent condition and two sufficient conditions in different forms for $\tilde{X}≽0$. We could now obtain QPSK candidate vectors $s_{can,k}^{\text{QPSK}},\;k=1,\;2,\cdots ,K$ and choose the one that maximizes the SCNR in Equation (19). We consider this subsection to be important work, because the QPSK signal generation technique is easy and mature, compared with arbitrary-phase or non-constant modulus signals. 

**Figure 2:**
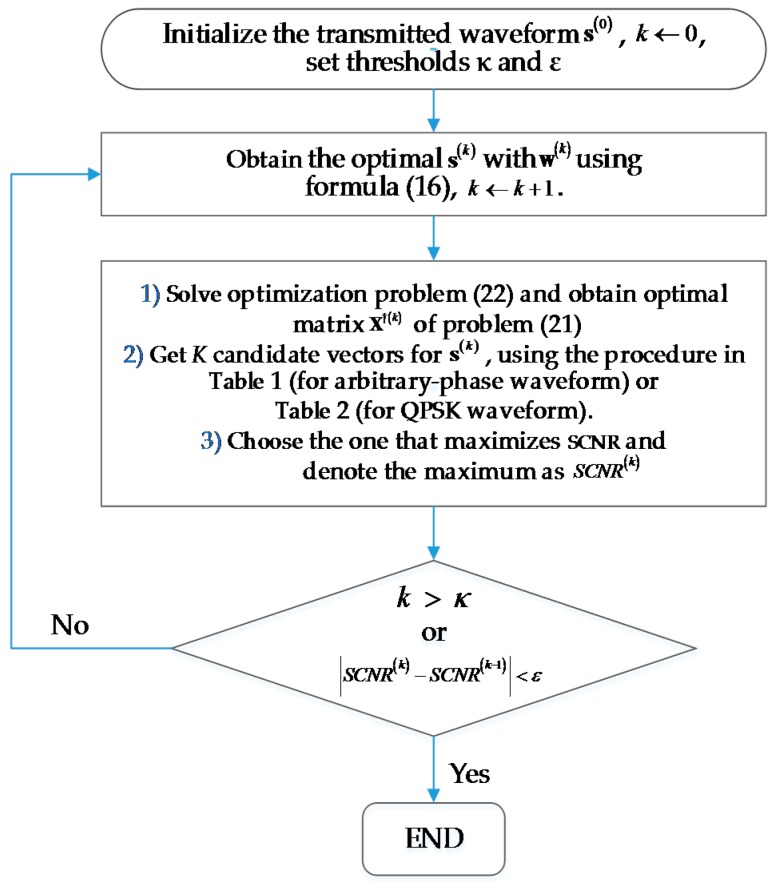
Flowchart of the proposed iterative constant-modulus waveform design methods.

Both our proposed iterative constant-modulus waveform design methods are summarized in Figure 2. We can see that the arbitrary-phase and QPSK waveform design methods share the same overall procedure, the only difference being the way in which candidate vectors are generated.

## 4. Algorithm Performance Analysis 

This section is composed of two parts: we first discuss further the performance loss caused by the used semi-definite relaxation; this discussion throws new insights into similar optimization problems; we then present an upper bound analysis of the SCNR increment at each iteration, an analysis that, in our opinion, is necessary for an iterative algorithm.

### 4.1. Semi-Definite Relaxation and Loss of Performance

In this subsection, we focus on the relationship between the original optimization problem Equation (19) and its corresponding SDR problem Equation (21). To ensure consistency with Section 3, $X^{†}$ still designates here the optimal solution to Equation (21) and could be obtained via the optimal solutions of Equation (22), $X_{opt}$ and $t_{opt}$. We denote the optimal solution of Equation (19) by $s_{opt}$, and the corresponding optimal SCNR by $u_{org}$, *i.e.*, $u_{org}=\left(s_{opt}^{H}\Omega_{1}s_{opt}\right)/\left(s_{opt}^{H}\Omega_{2}s_{opt}\right)$. For the sake of simplicity, we define

$$\begin{array}{l} u_{sdp}≜\frac{tr\left(\Omega_{1}X^{†}\right)}{tr\left(\Omega_{2}X^{†}\right)}\\ u\left(rd\right)≜\max\;\;\frac{s_{can,k}^{H}\;\Omega_{1}\;s_{can,k}}{s_{can,k}^{H}\;\Omega_{2}\;s_{can,k}},\;\;k=1,\;\cdots ,K\\ u\left(ep\right)≜\;E\left(\frac{s_{can,k}^{H}\;\Omega_{1}\;s_{can,k}}{s_{can,k}^{H}\;\Omega_{2}\;s_{can,k}}\right) \end{array}$$

where u(rd) andu(ep) represent the optimal and expected value of the SCNRs obtained from the candidate vectors, respectively. Based on our definitions, it is easily verified that

(28) $$u_{org}\geq u\left(rd\right)\geq u\left(ep\right)$$

We will later further discuss the relationship of $u_{org}$, $u_{sdp}$, u(rd) and  u(ep). The following lemma addresses the effect of modulus normalization $s_{\left(can,k\right)}=exp\left(-j\cdot ang\left(s_{\left(k\right)}\right)\right)$ on the signal covariance matrix.

**Lemma 2.**  *Assume that $X\in {\mathbb{C}}^{N\times N}$ is a Hermitian matrix satisfying X≽0 or X≻0, diag(X)=1 and $|X_{pq}|\leq 1$ for all 1≤p<q≤N. $\hat{s}$ denotes the unimodular random vector obtained from X with the randomization procedure of Table 1. Then we have*

(29) $$E\left(\hat{s}{\hat{s}}^{H}\right)=F\left(X\right)$$

*where F(·) is a component-wise matrix function*

$$F\left(X\right)={\left[F\left(X_{pq}\right)\right]}_{N\times N}$$

*and*

(30) $$F\left(x\right)=\frac{1}{4\pi }\int_{0}^{2\pi }e^{j\theta }{\left(\arccos\left(-\gamma \cos\left(\theta -\alpha \right)\right)\right)}^{2}d\theta$$

*where x∈ℂ with 0≤γ=|x|≤1 and α=arg(x).*

The proof of this Lemma is given in Appendix A. Lemma 2 provides the change of covariance matrix of the random vectors after modulus normalization. With the help of Lemma 2, we introduce the following theorem.

**Theorem 3.**  *For optimization problem Equations (19) and (21), it holds that*

(31) $$u_{org}\geq u\left(rd\right)\geq u\left(ep\right)\geq \frac{\pi u_{1}}{4u_{\max}}\cdot u_{org}$$

*where $u_{1}=tr\left(\Omega_{2}X^{†}\right)$, $u_{max}=\underset{k=0,1,2\cdots }{\sup}\;tr\left(\Omega_{2}\left({\left|X^{†}\right|}^{\left(2k\right)}\circ X^{†}\right)\right)$, ∘ is the Hadamard product operator, and the superscript (k) means*

$$A^{\left(k\right)}=\underset{k}{\underset{︸}{A\circ A\circ \cdots \circ A}}$$

*We define $A^{\left(0\right)}≜1_{N\times N}$ to make $\left({\left|X\right|}^{\left(2k\right)}\circ X\right)$ equal X when k=0*.

The proof of Theorem 3 can be found in Appendix B. Particularly, if $u_{\text{max}}=\text{tr}\left(\Omega_{2}\underset{k\to \infty }{\lim}\left({\left|X^{†}\right|}^{\left(2k\right)}\circ X^{†}\right)\right)$, and $X^{†}$ is a matrix satisfying $\left|X^{†}{}_{pq}\right|<1\;for\;1\leq p\neq q\leq N_{s}$, it follows that $\underset{k\to \infty }{\lim}\left({\left|X^{†}\right|}^{\left(2k\right)}\circ X^{†}\right)=I$ and $u_{\text{max}}=\text{tr}\left(\Omega_{2}\right)$. Therefore, we have

$$u\left(rd\right)\geq \frac{\pi u_{1}}{4tr\left(\Omega_{2}\right)}\cdot u_{org}$$

From the proof in Appendix B, we can see that the performance loss of Equation (21) relative to Equation (19), is directly related to the approximation to $X^{†}$ of the normalized unimodular vectors or QPSK vectors covariance matrix. The closer the approximation, the smaller the performance loss will be. As shown in [34], our QPSK signal design scheme could approximate the original matrix $X^{†}$ quite closely. We will not talk further about the QPSK signal case. However, it is still interesting to note that, according to [36], there will not be any performance loss between the optimization problem

(32) $$\underset{s}{\max}\frac{s^{H}\Omega_{1}s}{s^{H}\Omega_{2}s}\;\;\;s.t.\;\;\left|s\left(k\right)\right|\in \left\{1,\;j,\;-1,\;-j\right\},\;k=1,\;\cdots ,\;N_{s}$$

and the corresponding problem Equation (21) after relaxation followed by the discrete randomization approach presented in Equation (A2) with m=4.

### 4.2. Upper Bound Analysis of the SCNR Increment at Each Iteration

The alternate optimization scheme along the w and s dimensions guarantees that SCNR will not decrease as iterations progress. In this subsection, we present a preliminary upper bound analysis of the SCNR increment at each iteration (A loose lower bound can be taken to be 0.). Noticing that, at every iteration, we solve Equation (15) and obtain the optimal SCNR $\lambda_{1}\left({\left(R_{cs}+R_{n}\right)}^{-1}R_{ts}\right)$, we just need to concentrate on $\lambda_{1}\left({\left(R_{cs}+R_{n}\right)}^{-1}R_{ts}\right)$ and analyze its increment. To that end, we first introduce the following lemma.

**Lemma 4.**  *Suppose that $A\in {\mathbb{C}}^{N\times N}=Q\Lambda Q^{-1}$ is diagonalizable and Q is an invertible matrix, Λ is diagonal, and $\Delta A,\;B\in {\mathbb{C}}^{N\times N}$ satisfy ΔA=B−A. Then for any $\mu \in \left\{\lambda_{i}\left(B\right),i=1,\cdots ,N\right\}$, there exists $\chi \in \left\{\lambda_{i}\left(A\right),i=1,\cdots ,N\right\}$ such that*

(33) $$\left|\chi -\mu \right|\leq \underset{p\geq 1}{\inf}\left[\mathrm{cond}\left(Q,p\right){\left\|\Delta A\right\|}_{p}\right]$$

*where ${∥\cdot ∥}_{p},\;p\geq 1$ represents the p-norm of a matrix, and $cond\left(Q,p\right)=∥Q^{-1}{∥}_{p}{∥Q∥}_{p}$ is generally called the condition number.*

The proof of Lemma 4 is similar with that of Theorem 3 in [37], except for extending to arbitrary *p*-norm (p≥1). Thus, Lemma 4 can be viewed as a straightforward extension of Theorem 3 in [37]. One can refer to [37] for the proof details. From Lemma 4, we can see that the upper bound of the change of the largest eigenvalue of matrix A due to the perturbation ΔA, *i.e.*, $\left|\lambda_{1}\left(A+\Delta A\right)-\lambda_{1}\left(A\right)\right|$, depends on the norm of ΔA and the condition number of matrix Q, whose columns are the eigenvectors of A.

For simplicity, we assume that $R_{ts}$ is full rank. The situation when $R_{ts}$ is rank deficient would not be much different. Given that $R_{ts}≻0$, there must exist an invertible matrix $M\in {\mathbb{C}}^{N\times N}$ such that $R_{ts}=MM^{H}$. Considering that $M^{H}{\left(R_{cs}+R_{n}\right)}^{-1}R_{ts}{\left(M^{H}\right)}^{-1}=M^{H}{\left(R_{cs}+R_{n}\right)}^{-1}M$, ${\left(R_{cs}+R_{n}\right)}^{-1}R_{ts}$ has the same eigenvalues as matrix $M^{H}{\left(R_{cs}+R_{n}\right)}^{-1}M$, which is Hermitian and positive definite. Note that $M^{H}{\left(R_{cs}+R_{n}\right)}^{-1}M$ is unitary similar to a diagonal matrix, *i.e.*,

(34) $$M^{H}{\left(R_{cs}+R_{n}\right)}^{-1}M=P\Lambda P^{-1}$$

where P is a unitary matrix, and Λ is diagonal with positive diagonal elements. One simple fact is that the unitary matrix P satisfies $∥P^{-1}{∥}_{2}{∥P∥}_{2}=1$. To simplify the exposition, we denote Equation (34) by Θ(s). Therefore, if one iteration transforms the transmitted waveform from s to s+Δs, the upper bound of the SCNR increment in this iteration is $∥\Theta \left(s+\Delta s\right)-\Theta {\left(s\right)∥}_{2}$, *i.e.*,

(35) $$\left|\lambda_{1}\left(\Theta \left(s+\Delta s\right)\right)-\lambda_{1}\left(\Theta \left(s\right)\right)\right|\leq {\left\|\Theta \left(s+\Delta s\right)-\Theta \left(s\right)\right\|}_{2}$$

Note that Θ(s) is a continuous function of s. Therefore, as Δs→0, the variation of Θ(s) also tends to 0, $∥\Theta \left(s+\Delta s\right)-\Theta {(s)∥}_{2}\to 0$, and finally $\left|\lambda_{1}\left(\Theta \left(s+\Delta s\right)\right)-\lambda_{1}\left(\Theta \left(s\right)\right)\right|\to 0$. This is sensible, and gives some insights into the rationality of Equation (35).

Also note that it is reasonable to state that the waveform design methods developed herein could be instrumental for improving the performance of the ultra-wide band cognitive radio [38] and sensor radar networks [39,40], considering that they share common issues such as clutter suppression, and extended target feature extraction. The adaption of the proposed technique to these scenarios is currently being investigated. 

## 5. Results

In this section, we demonstrate the effectiveness of the proposed methods. The simulation parameters are as follows. The length of $h_{t}$ is $N_{t}=20$, while the phase coding length of the transmitted waveform is $N_{s}=50$. Without any loss of generality, we let the noise covariance matrix be $R_{n}=\sigma_{n}^{2}I$, with $\sigma_{n}^{2}=0.01$. The sample number in the randomization approach is *K* = 10,000. As in [26], we use a random phase-coded signal as the initial transmitted waveform. $R_{c}$ is modeled as being the covariance matrix of the output of a linear time invariant (LTI) filter with impulse response h, whose input signal is complex Gaussian white noise with unit variance. h is assumed to be:

$$h\left(n\right)=\left\{ \begin{array}{l} 1,\;\;\;n=0,\;1,\;\cdots ,\;14\\ 0,\;\;\;\;otherwise \end{array}\right.$$

**Figure 3:**
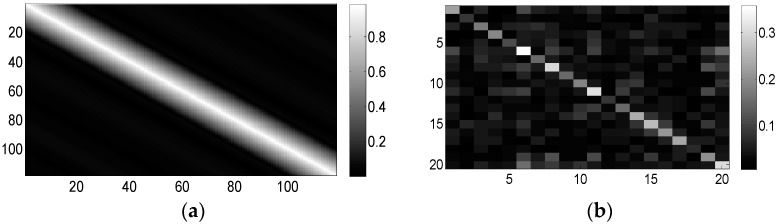
Illustration of (**a**) matrix $R_{c}$, the covariance matrix of the clutter impulse response, $h_{c}$; (**b**) $R_{t}$, the covariance matrix of target impulse response $h_{t}$, used in Section 5.1.

The element values of $R_{c}\;$are illustrated graphically in Figure 3a. We denote the matrix in Figure 3a by $R_{c}^{\left(1\right)}$ and further let $R_{c}\;$= $\sigma_{c}^{2}R_{c}^{\left(1\right)}$, where $\sigma_{c}^{2}$ can be considered to be the clutter power. Thereby, we define the clutter-to-noise ratio (CNR) as $\sigma_{c}^{2}/\sigma_{n}^{2}$. Without specific statements, CNR is set to 10 dB. The iteration number starts at 1 and increases every time we compute Equation (16).

We will now test and verify the performances of the proposed Algorithms 1 and 2 in two cases: the statistical target case and the deterministic target case.

### 5.1. Statistical Target Case

**Figure 4:**
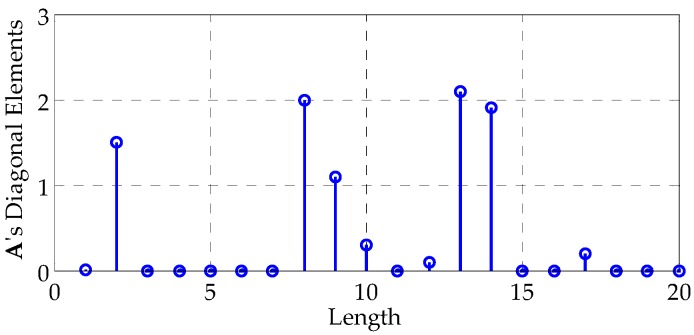
Illustration of the diagonal elements of matrix **A**.

In this subsection, we consider a statistical $h_{t}$. To guarantee positive semi-definiteness, we generate $R_{t}$ using

$$R_{t}=AA^{H}$$

where A is a matrix whose elements are generated as independent and identically distributed (i.i.d.) circular complex Gaussian random variables with unit variance, except for the diagonal elements, which are shown in Figure 4. Note that this approach to generate $R_{t}$ is similar to the one in [26]. The considered target has five significant reflection centers and is similar to the SR-71 aircraft model [25]. Figure 3b illustrates the elements in $R_{t}$. The diagonal elements of A (and $R_{t}$) can naturally be chosen differently. The parameters chosen here are simply a common example.

As a contrast, we also present the waveform design technique with the total energy constraint proposed in [26]—which also cyclically optimizes the transceiver pair, but uses Equation (18) to compute **s** in terms of **w**—instead of solving the complicated optimization problems posed by Equation (19) or (21). Consequently, it cannot guarantee the constant modulus property, which hampers the practicability of the designed waveforms. Nevertheless, the absence of the constant modulus constraint leads to a better SCNR performance with extra degrees of freedom. Additionally, to highlight the advantages of our optimization technique, we define the following discrete LFM signal:

(36) $$s_{LFM}\left(n\right)=\exp\left(j\cdot k\pi {\left(\frac{n-1}{N_{t}}\right)}^{2}\right),\;\;\;n=1,2,\cdots ,N_{s}$$

We take k=20 in our simulation. Correspondingly, we set the vector w obtained with Equation (16) as the matched filter for $s_{LFM}$. 

**Figure 5:**
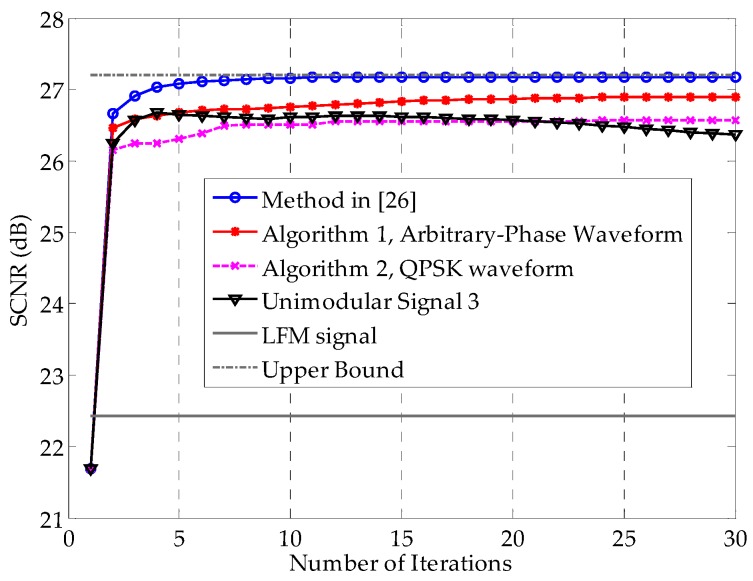
SCNR performances as a function of the number of iterations for different methods. The initial waveforms are identical for all methods. Algorithms 1 and 2 are the arbitrary-phase and QPSK signal design methods proposed in Section 3, respectively. Unimodular Signal 3 denotes the unimodular signal with the phase that results from the method in [26], *i.e.*, a modulus normalized version of the waveform obtained under the total energy constraint. The upper bound is the limiting value of the SCNR obtained using the method in [26]. The SCNR of the LFM signal does not vary with the iterations, because this signal is fixed.

**Figure 6:**
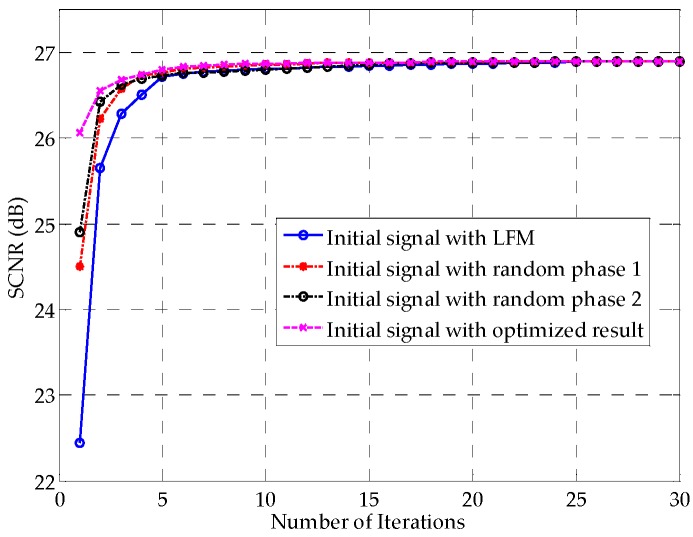
SCNR performances *vs.* the number of iterations for different initial waveforms.

Figure 5 shows the SCNR performances as a function of the number of iterations. The method in [26], as was expected, converges to the best SCNR among these methods, benefiting from the additional degree of freedom offered by the envelope flexibility. Note that Algorithm 1 also converges fast and has only a 0.28 dB SCNR performance loss compared with the method in [26] after convergence. Generally speaking, with the benefit of the constant modulus property, the SCNR loss of 0.28 dB is tolerable. Compared with the method in [26], Algorithm 2 shows a 0.61 dB SCNR loss, with improved implementation. The Unimodular Signal 3, which uses the phases of the results of method in [26], requires fewer computations, without any convex optimization problems to solve. However, its performance cannot be trusted, even though it performs moderately in this example. As Figure 5 shows, it might end up with a worse result than Algorithm 2. Nevertheless, it also implies that a unimodular signal with the phases of the first iteration’s result using the method in [26] is a reasonable initial waveform for Algorithm 1. Figure 6 verifies this notion. Among the four initially used waveforms, the one with the preliminary optimized result of the method in [26]—plotted with the red dashed line—converges the fastest. The LFM signal represented in Equation (36) has the worst initial SCNR and converges the slowest in this example, revealing that the LFM signal is not as advantageous for extended targets as it is for point targets.

**Figure 7:**
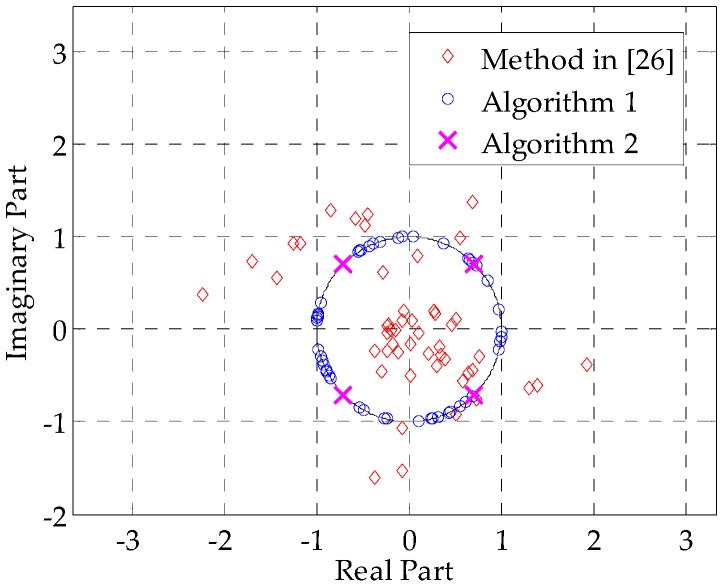
Real and imaginary parts of different methods’ results.

Figure 7 presents the real and imaginary parts of the optimized signals obtained by different methods. One can clearly see that the method in [26]—marked by red diamonds—cannot guarantee the constant modulus property of the designed signal. In contrast, the results of Algorithm 1 are unimodular and lie on the unit circle, whereas the results of Algorithm 2 are fixed to four phase points, which validates our constraints for the constant-modulus signal and QPSK signal.

**Figure 8:**
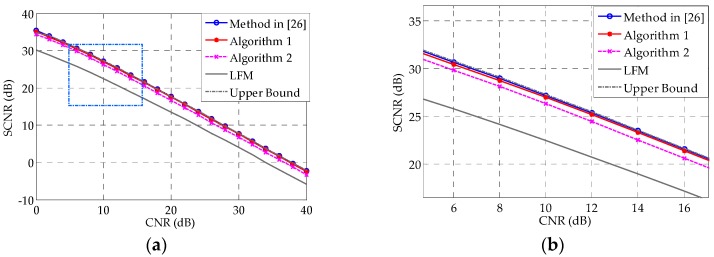
(**a**) Comparison of the SCNR of different methods *vs.* CNR. The upper bound is the SCNR obtained with 100 iterations of the method in [26], while the blue line marked with circles is obtained with 30 iterations of the same method; (**b**) Zoomed version of the boxed region in (**a**).

Figure 8 shows the SCNR performances of different methods, as a function of CNR. It is intuitive that, as the CNR increases, the SCNR performance worsens correspondingly. One can see that Algorithms 1 and 2 can closely approximate the upper bound over a large range of CNRs. Compared with the LFM signal in Figure 8, our optimization technique could achieve approximately a 5 dB SCNR gain.

### 5.2. Deterministic Target Case

In this subsection, we assume that the TIR $h_{t}$ is deterministic and known. For demonstration, $h_{t}$ is set as the sequence shown in Figure 4. In this case, $R_{t}=h_{t}h_{t}{}^{H}$ is a rank-one matrix. The other parameters remain the same as in Section 5.1. We now briefly introduce a discrete-time version of the *eigen-iterative* algorithm in [22]. This algorithm was not discussed in Section 5.1, because it is not easily extended to a statistical target case. In this case of a deterministic and known TIR, the SCNR expression is reduced from Equation (8) to

(37) $$SCNR=\frac{{\left|w^{H}Ts\right|}^{2}}{w^{H}\left(R_{cs}+R_{n}\right)w},\;\;T=f\left(h_{t},N_{s}\right)$$

where function f(·) is defined in Section 2. Similar with Equation (15), the optimal receiving filter in terms of **s** is

(38) $$w_{opt}={\left(R_{cs}+R_{n}\right)}^{-1}Ts$$

Substituting Equation (38) into Equation (37) yields:

(39) $$\underset{s}{SCNR}=s^{H}T^{H}{\left(R_{cs}+R_{n}\right)}^{-1}Ts$$

To maximize the new objective function in Equation (39), it seems that we just need to take **s** as:

(40) $$v_{1}\left(T^{H}{\left(R_{cs}+R_{n}\right)}^{-1}T\right)$$

**Figure 9:**
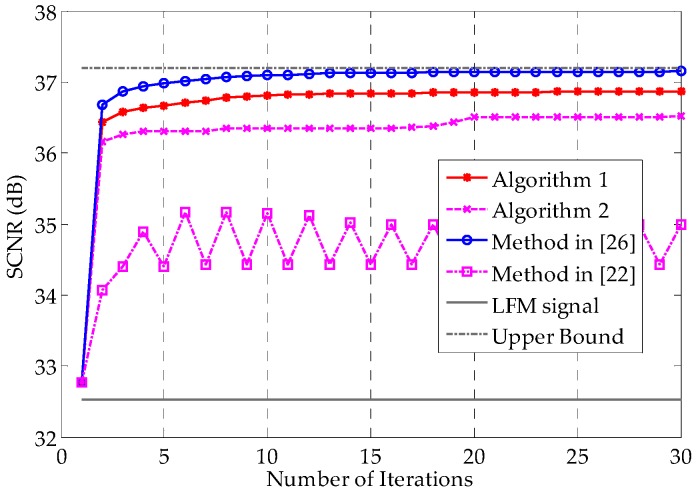
SCNR performances as a function of the number of iterations for the deterministic target case. The initial waveforms are identical for all methods.

However, the matrix in Equation (40) is also a function of **s**. The authors in [22] proposed to cyclically update **s** to approach the optimally transmitted waveform using Equation (40). However, as pointed out in [26], the method in [22] cannot ensure performance improvements as iterations go on. Also note that the method cannot achieve the largest eigenvalue of $T^{H}{\left(R_{cs}+R_{cs}\right)}^{-1}T$ as its output SCNR, because the signal **s** obtained with Equation (40), which would be the waveform for the next cycle, is not the **s** in Equation (39). Figure 9 shows the SCNR performances as a function of the iteration index in the deterministic target case. One can see that Algorithms 1 and 2 are well suited for the deterministic target case. The optimized QPSK signal performed better than both the method in [22] and LFM signal, even though it can only choose from four phase points. Compared with other methods, the method in [22] suffers from performance degradation and fluctuations. As mentioned earlier, the LFM signal in Equation (36) does not perform well in the deterministic extended target, and performed even worse than the initial random-phase signal in this example.

**Figure 10:**
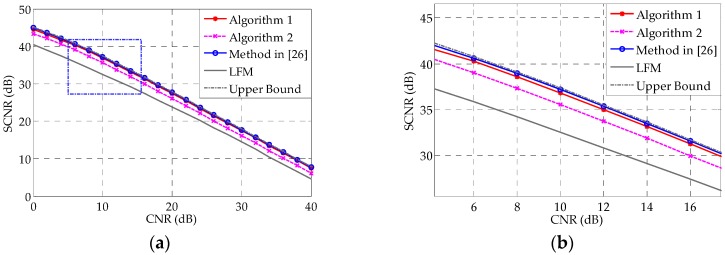
(**a**) SCNR performances *vs.* CNR for the deterministic target case. The upper bound is the SCNR obtained with 100 iterations of the method in [26], while the blue line marked with circles is obtained with 30 iterations of the same method; (**b**) Zoomed version of the box highlighted in (**a**).

Figure 10 shows the SCNR performances as functions of CNR in the range 0–40 dB. We do not include the method in [22] in this example, because it did not converge. It can be seen that Algorithms 1 and 2 can closely approximate the method in [26] for a large range of CNRs, even though they are restricted to constant modulus—once again demonstrating the effectiveness of the proposed methods.

## 6. Conclusions

In this paper, we proposed two iterative constant-modulus waveform design algorithms for the detection of extended targets, with prior information of the target and waveform-dependent clutter. To deal with the intractable problem brought by the constant modulus constraint, we made use of a semi-definite relaxation approach and developed a customized randomization method. Moreover, combining this with our previous work, we further advanced the design of QPSK waveforms. We then discussed the relationship between the nonconvex problem and its corresponding convex form after SDR, and analyzed an upper bound on the SCNR increment at each iteration. Even though our methods were established based on a statistical target model, they can be applied to the deterministic target case as well. The obtained numerical results show that the proposed algorithms have satisfied SCNR performances in both the statistical and deterministic target cases. However, as mentioned earlier, the alternate optimization technique cannot guarantee the convergence to the global optimum. The probability of this technique getting trapped in a local optimum and the search for a better optimization strategy are open for further study. Additionally, we assumed that the statistical characteristics of the target and clutter are known. The effective estimation of these prior information from radar returns is therefore well worth exploring. We also note that the final waveforms obtained with the proposed methods may not have good performance concerning range resolution. Waveform design taking range resolution into account will also be investigated in the future.

## Figures and Tables

**Table 1:** Customized randomization approach procedure.

**If** $\text{rank}\left(X_{opt}\right)=1$ $s_{opt}$ is the first row of matrix $X^{†}$; **Otherwise** *Step 1*: Obtain $X^{†}=U\Sigma U^{H}$ from the eigenvalue decomposition of $X^{†}$. *Step 2*: Generate *K* independent random vectors $v_{\left(k\right)},k=1,\;2,\cdots ,K$, from complex-valued Gaussian distribution CN(0,I). *Step 3*: Compute $s_{\left(k\right)}=U\Sigma^{1/2}v_{\left(k\right)},k=1,\;2,\cdots ,K$ so that the covariance matrix of $s_{\left(k\right)}$ is $X^{†}$. *Step 4*: Normalize the modulus of $s_{k}$: $s_{\left(can,k\right)}=exp\left(-j\cdot ang\left(s_{\left(k\right)}\right)\right),k=1,\;2,\cdots ,K$. ($s_{\left(can,k\right)}$ is a candidate vector for $s_{opt}$.) *Step 5*: Choose the candidate vector that maximizes the SCNR, *i.e.*, $$s_{opt}=\arg\;\underset{s_{\left(can,k\right)}}{\max}\;\frac{s_{\left(can,k\right)}^{H}\;\Omega_{1}\;s_{\left(can,k\right)}}{s_{\left(can,k\right)}^{H}\;\Omega_{2}\;s_{\left(can,k\right)}},\;\;k=1,\;\cdots ,K.$$

**Table 2:** Algorithm 1 full procedure.

*Step 0*: Initialize the transmitted signal $s^{\left(0\right)}$ with linear frequency modulation (LFM) signal or a random phase-coded signal. *Step 1*: Compute $R_{ts}$ with Equation (10), and obtain the receiving filter w using Equation (16) *Step 2*: Compute $R_{tw}$ with Equation (13), and obtain the optimal solution $X^{†}$ of Equation (21). Use the preceding randomization approach in Table 1 to get $s_{opt}$. *Step 3*: Go back to Step 1 unless the SCNR improvement becomes insignificant or iterative number becomes large enough.

**Table 3:** Candidate quadrature phase-shift keying (QPSK) signal vectors design procedure.

*Step 1*: Denote the real part and the imaginary part of $X^{†}$ by $X_{R}$ and $X_{I}$, respectively. *Step 2*: Generate matrix $\tilde{X}$ using (27) $$\tilde{X}=\left[\begin{array}{cc} A & B^{T}\\ B & A \end{array}\right],\;\;A=\sin\left(\frac{\pi }{2}X_{R}\right),\;B=\sin\left(\frac{\pi }{2}X_{I}\right)$$ where the sine function operates element-wise: $\sin\left(A\right)={\left[\sin\left(A_{mn}\right)\right]}_{N_{s}\times N_{s}}$. *Step 3*: Make a forced positive definite Cholesky decomposition $\tilde{X}+D=\Gamma \Gamma^{T}$, where **D** is a diagonal matrix with nonnegative elements. *Step 4*: Let β=Γθ, where θ~CN(0,I), *i.e.*, θ is normally distributed with zero mean. The QPSK signal can then be generated by $s=sgn\left(\beta \left(1:N_{s}\right)\right)+j\cdot sgn\left(\beta \left(N_{s}+1:2N_{s}\right)\right)$.

## Appendix A

**Proof of Lemma 2.** Firstly, we consider the discrete situation by defining

(A1) Fm(z)≜m(2−ω−1−ω)8π2∑n=0m−1ωn(arccos(−Re(ω−nz)))2

and assigning

(A2) $${\hat{z}}_{k}≜\sigma \left(s_{k}\right)=\left\{ \begin{array}{l} 1,\;\;\;\;\;\;\;\;\;if\;\arg\left(s_{k}\right)\in \left[0,\;\frac{2\pi }{m}\right)\\ \vdots \\ \omega^{l},\;\;\;\;\;\;if\;\arg\left(s_{k}\right)\in \left[\frac{l}{m}2\pi ,\;\frac{l+1}{m}2\pi \right)\\ \vdots \\ \omega^{m-1},\;\;\;\;if\;\arg\left(s_{k}\right)\in \left[\frac{m-1}{m}2\pi ,\;2\pi \right) \end{array}\right.$$

where s ~ CN(0,X), m≥2 is a given positive integer, and $\omega =exp\left(j\frac{2\pi }{m}\right)$. Supposing that $X_{pq}=\gamma e^{j\alpha },\;1\leq p,q\leq N$, we have

(A3) $$\begin{array}{l} \;\;\;\mathrm{Pr}\left({\hat{z}}_{p}{\hat{z}}_{q}^{*}=\omega^{n}\right)\\ =\;\;\sum_{l=0}^{m-1}\mathrm{Pr}\left({\hat{z}}_{p}=\omega^{l+n},\;{\hat{z}}_{q}=\omega^{l}\right)\\ =\;\;\sum_{l=0}^{m-1}\mathrm{Pr}\left(\arg\left(s_{p}\right)\in \left[\frac{l+n}{m}2\pi ,\;\frac{l+n+1}{m}2\pi \right),\;\arg\left(s_{q}\right)\in \left[\frac{l}{m}2\pi ,\;\frac{l+1}{m}2\pi \right)\right)\\ =\;\;\sum_{l=0}^{m-1}\left[\frac{1}{m^{2}}+\frac{1}{8\pi^{2}}\left(\begin{array}{l} 2\arccos{\left(-\gamma \cos\left(\frac{n}{m}2\pi +\alpha \right)\right)}^{2}-\arccos{\left(-\gamma \cos\left(\frac{n-1}{m}2\pi +\alpha \right)\right)}^{2}\\ -\arccos{\left(-\gamma \cos\left(\frac{n+1}{m}2\pi +\alpha \right)\right)}^{2} \end{array}\right)\right]\\ =\;\;\frac{1}{m}+\frac{m}{8\pi^{2}}\left(\begin{array}{l} 2\arccos{\left(-\gamma \cos\left(\frac{n}{m}2\pi +\alpha \right)\right)}^{2}-\arccos{\left(-\gamma \cos\left(\frac{n-1}{m}2\pi +\alpha \right)\right)}^{2}\\ -\arccos{\left(-\gamma \cos\left(\frac{n+1}{m}2\pi +\alpha \right)\right)}^{2} \end{array}\right) \end{array}$$

where Pr(A) denotes the probability that A holds, and the third equality utilizes Theorem 2.5 in [36], concerning complex bivariate normal distributions. Furthermore, we have

(A4) E[z^pz^q∗]=  ∑n=0m−1ωnPr(z^pz^q∗=ωn)=  m8π2∑n=0m−1ωn(2arccos(−γcos(nm2π+α))2−arccos(−γcos(n−1m2π+α))2−arccos(−γcos(n+1m2π+α))2)=  m8π2∑n=0m−1(2ωn−ωn−1−ωn+1)arccos(−γcos(nm2π+α))2=  m(2−ω−1−ω)8π2∑n=0m−1ωnarccos(−γcos(nm2π+α))2=  m(2−ω−1−ω)8π2∑n=0m−1ωnarccos(−Re(ωnXpq))2

Notice that the conjugation of $F_{m}\left(X_{pq}\right)$ satisfies

(A5) $$\begin{array}{l} F_{m}^{*}\left(X_{pq}\right)\\ =\frac{m\left(2-\omega^{-1}-\omega \right)}{8\pi^{2}}\sum_{n=0}^{m-1}\omega^{-n}{\left(\arccos\left(-\gamma \cos\left(-\frac{n}{m}2\pi +\alpha \right)\right)\right)}^{2}\\ =\frac{m\left(2-\omega^{-1}-\omega \right)}{8\pi^{2}}\sum_{n=0}^{m-1}\omega^{m-n}{\left(\arccos\left(-\gamma \cos\left(\frac{m-n}{m}2\pi +\alpha \right)\right)\right)}^{2}\\ =\frac{m\left(2-\omega^{-1}-\omega \right)}{8\pi^{2}}\sum_{n=0}^{m-1}\omega^{n}{\left(\arccos\left(-\gamma \cos\left(\frac{n}{m}2\pi +\alpha \right)\right)\right)}^{2}\\ =E\left[{\hat{z}}_{p}{\hat{z}}_{q}^{*}\right] \end{array}$$

In accordance with Table 1, the *k*th element of $\hat{s}$ satisfies ${\hat{s}}_{k}={\left({\hat{z}}_{k}\right)}^{*},\;k=1,\cdots ,N$. Thus, we have that

(A6) $$E\left[{\hat{s}}_{p}{\hat{s}}_{q}^{*}\right]={\left(E\left[{\hat{z}}_{p}{\hat{z}}_{q}^{*}\right]\right)}^{*}=F_{m}\left(X_{pq}\right)$$

For the discrete case, the covariance matrix of the random vector $\hat{s}$ is

(A7) $$E\left[\hat{s}{\hat{s}}^{H}\right]=E{\left[\hat{z}{\hat{z}}^{H}\right]}^{*}=F\left(X\right)$$

which holds for any integer m≥2. For a given $z=\gamma e^{j\alpha }\in \mathbb{C}$ with 0≤γ≤1, it holds that

(A8) Fm(z)=  m(2−ω−1−ω)8π2∑n=0m−1ωn(arccos(−Re(ω−nz)))2=  m28π3(1−cos(2πm))⋅∑n=0m−1[ωn(arccos(−γcos(nm2π−α)))2⋅2πm] 

Taking the limit m→∞, we get

(A9) $$F\left(z\right)=\underset{m\to \infty }{\lim}F_{m}\left(z\right)=\frac{1}{4\pi }\int_{0}^{2\pi }e^{j\theta }{\left(\arccos\left(-\gamma \cos\left(\theta -\alpha \right)\right)\right)}^{2}d\theta$$

where the equation

(A10) $$\underset{m\to \infty }{\lim}m^{2}\left(1-\cos\left(\frac{2\pi }{m}\right)\right)=2\pi^{2}$$

is used. This completes the proof.

## Appendix B

**Proof of Theorem 3.** Define $\hat{X}=s_{opt}s_{opt}^{H}$. $\hat{X}$ is easily verified to be a feasible solution of Equation (21). Because of the optimality of $X^{†}$, we have

(B1) $$u_{sdp}=\frac{tr\left(\Omega_{1}X^{†}\right)}{tr\left(\Omega_{2}X^{†}\right)}\geq \frac{tr\left(\Omega_{1}\hat{X}\right)}{tr\left(\Omega_{2}\hat{X}\right)}=u_{org}$$

For notational simplicity, we use Z to indicate $X^{†}$ in the following proof procedure. One can easily check that Z satisfies the restrictions of Lemma 2. Suppose that the randomization approach with Z yields a candidate vector $\hat{s}$. Then we have

(B2) $$E\left(\hat{s}{\hat{s}}^{H}\right)=F\left(Z\right)$$

We now consider the relationship between Z and F(Z). For $z=\gamma e^{j\alpha }\in \mathbb{C}$ with 0≤γ≤1, we have

(B3) $$\begin{array}{l} F\left(z\right)\;=\;\;\frac{1}{4\pi }\int_{0}^{2\pi }e^{j\theta }{\left(\arccos\left(-\gamma \cos\left(\theta -\alpha \right)\right)\right)}^{2}d\theta \\ \;\;\;\;\;\;\;=\;\;\frac{1}{4\pi }e^{j\alpha }\left[\int_{0}^{\pi }e^{j\theta }{\left(\arccos\left(-\gamma \cos\theta \right)\right)}^{2}d\theta -\int_{0}^{\pi }e^{j\theta }{\left(\arccos\left(\gamma \cos\theta \right)\right)}^{2}d\theta \right]\\ \;\;\;\;\;\;\;=\;\;\frac{1}{2}e^{j\alpha }\int_{0}^{\pi }e^{j\theta }\arcsin\left(\gamma \cos\theta \right)d\theta \\ \;\;\;\;\;\;\;=\;\;\frac{1}{2}e^{j\alpha }\int_{0}^{\pi }e^{j\theta }\left(\gamma \cos\theta +\sum_{k=1}^{\infty }\frac{\left(2k\right)!}{4^{k}{\left(k!\right)}^{2}\left(2k+1\right)}{\left(\gamma \cos\theta \right)}^{2k+1}\right)d\theta \\ \;\;\;\;\;\;\;=\;\;\frac{\pi }{4}\gamma e^{j\alpha }+\frac{\pi }{2}\sum_{k=1}^{\infty }\frac{{\left(\left(2k\right)!\right)}^{2}}{2^{4k+1}{\left(k!\right)}^{4}\left(k+1\right)}\gamma^{2k+1}e^{j\alpha }\\ \;\;\;\;\;\;\;=\;\;\frac{\pi }{4}z+\frac{\pi }{2}\sum_{k=1}^{\infty }\frac{{\left(\left(2k\right)!\right)}^{2}}{2^{4k+1}{\left(k!\right)}^{4}\left(k+1\right)}{\left|z\right|}^{2k}z\; \end{array}$$

where the third equality exploits that arccos(−γcosθ)=π−arccos(γcosθ), the next to last equality results from the two following identities:

$$\int_{0}^{\pi }\sin\theta {\left(\cos\theta \right)}^{2k+1}d\theta =0,\;\;\int_{0}^{\pi }{\left(\cos\theta \right)}^{2k+2}d\theta =\frac{\left(2k\right)!\left(2k+1\right)}{2^{2k+1}{\left(k!\right)}^{2}\left(k+1\right)}\pi$$

Thus,

(B4) $$F\left(Z\right)=\frac{\pi }{4}Z+\frac{\pi }{2}\sum_{k=1}^{\infty }\frac{{\left(\left(2k\right)!\right)}^{2}}{2^{4k+1}{\left(k!\right)}^{4}\left(k+1\right)}{\left|Z\right|}^{\left(2k+1\right)}\circ Z$$

Subsequently, we introduce one simple fact about the Hadamard product [41]—for two positive semi-definite matrices A,B, A∘B remains positive semi-definite. Considering that

$${\left|Z\right|}^{\left(2k\right)}=\underset{k}{\underset{︸}{\left(Z\circ Z^{T}\right)\circ \cdots \circ \left(Z\circ Z^{T}\right)}}$$

We have ${\left|Z\right|}^{\left(2k\right)}\circ Z≽0$, further yielding $F\left(Z\right)≽\frac{\pi }{4}Z$, and

(B5) $$tr\left(\Omega_{1}F\left(Z\right)\right)\geq \frac{\pi }{4}\cdot tr\left(\Omega_{1}Z\right)$$

Because $\left|Z_{pq}\right|\leq 1$ for $1\leq p,q\leq N_{s}$, the limit of $\underset{k\to \infty }{\lim}{\left|Z\right|}^{\left(2k\right)}\circ Z$ exists and is denoted as $\bar{Z}$, which is a matrix whose diagonal elements are all 1 and all other elements have either 0 or 1 modulus. Hence, $\underset{k\to \infty }{\lim}tr\left(\Omega_{2}\left({\left|Z\right|}^{\left(2k\right)}\circ Z\right)\right)=tr\left(\Omega_{2}\bar{Z}\right)$. A supremum for sequence $tr\left(\Omega_{2}\left({\left|Z\right|}^{\left(2k\right)}\circ Z\right)\right),\;$k=0, 1,⋯ must exist; let us denote it by $u_{max}$; therefore,

(B6) $$tr\left(\Omega_{2}\left({\left|Z\right|}^{\left(2k+1\right)}\circ Z\right)\right)\leq u_{\max},\;for\;k=0,\;1,\;\cdots$$

Note that both the diagonal elements of F(X) and of the covariance matrix of the unimodular vector $\hat{s}$ are 1. Therefore, Equation (29) in Lemma 2 implies that F(1)=1, *i.e.*,

$$\frac{\pi }{4}+\frac{\pi }{2}\sum_{k=1}^{\infty }\frac{{\left(\left(2k\right)!\right)}^{2}}{2^{4k+1}{\left(k!\right)}^{4}\left(k+1\right)}=1$$

Hence, it holds that

(B7) $$\begin{array}{l} \;\;\;\;\;tr\left(\Omega_{2}F\left(Z\right)\right)\\ =\;\;\;\frac{\pi }{4}tr\left(\Omega_{2}Z\right)+\frac{\pi }{2}\sum_{k=1}^{\infty }\frac{{\left(\left(2k\right)!\right)}^{2}}{2^{4k+1}{\left(k!\right)}^{4}\left(k+1\right)}tr\left(\Omega_{2}\left({\left|Z\right|}^{\left(2k+1\right)}\circ Z\right)\right)\\ \leq \;\;\;\left(\frac{\pi }{4}+\frac{\pi }{2}\sum_{k=1}^{\infty }\frac{{\left(\left(2k\right)!\right)}^{2}}{2^{4k+1}{\left(k!\right)}^{4}\left(k+1\right)}\right)u_{\max}\;\;=\;\;\;u_{\max} \end{array}$$

Therefore,

(B8) $$tr\left(\Omega_{2}F\left(Z\right)\right)\leq \frac{u_{\max}}{u_{1}}tr\left(\Omega_{2}Z\right)$$

Combining Equations (B5) and (B8) yields

(B9) $$\frac{tr\left(\Omega_{1}F\left(Z\right)\right)}{tr\left(\Omega_{2}F\left(Z\right)\right)}\geq \frac{\pi u_{1}}{4u_{\max}}\cdot \frac{tr\left(\Omega_{1}Z\right)}{tr\left(\Omega_{2}Z\right)}$$

Using Equation (B2), one can easily get

(B10) $$u\left(ep\right)=E\left(\frac{tr\left({\hat{s}}^{H}\Omega_{1}\hat{s}\right)}{tr\left({\hat{s}}^{H}\Omega_{2}\hat{s}\right)}\right)=\frac{tr\left(\Omega_{1}E\left(\hat{s}{\hat{s}}^{H}\right)\right)}{tr\left(\Omega_{2}E\left(\hat{s}{\hat{s}}^{H}\right)\right)}=\frac{tr\left(\Omega_{1}F\left(Z\right)\right)}{tr\left(\Omega_{2}F\left(Z\right)\right)}$$

The maximum value of the random variable $\frac{tr\left({\hat{s}}^{H}\Omega_{1}\hat{s}\right)}{tr\left({\hat{s}}^{H}\Omega_{2}\hat{s}\right)}$, u(rd), must be larger than its expected value, u(ep), when K is large enough, *i.e.*,

(B11) u(rd)≥u(ep)

Simultaneous consideration of Equations (28) and (B9)–(B11) produces Equation (31). This then concludes the proof.
